# Peripheral Mechanisms Underlying Bacillus Calmette–Guerin-Induced Lower Urinary Tract Symptoms (LUTS)

**DOI:** 10.3390/brainsci14121203

**Published:** 2024-11-28

**Authors:** Meera Elmasri, Aaron Clark, Luke Grundy

**Affiliations:** Flinders Health and Medical Research Institute (FHMRI), College of Medicine and Public Health, Flinders University, Adelaide 5042, Australia; elma0018@flinders.edu.au (M.E.); clar1171@flinders.edu.au (A.C.)

**Keywords:** bladder cancer (BCa), NMIBC, Bacillus Calmette–Guérin (BCG), cystitis, LUTSs, afferent sensitisation

## Abstract

Non-muscle invasive bladder cancer (NMIBC) accounts for approximately 70–75% of all bladder cancer cases. The standard treatment for high-risk NMIBC involves transurethral tumour resection followed by intravesical Bacillus Calmette–Guerin (BCG) immunotherapy. While BCG immunotherapy is both safe and effective, it frequently leads to the development of lower urinary tract symptoms (LUTS) such as urinary urgency, frequency, dysuria, and pelvic discomfort. These symptoms can significantly diminish patients’ quality of life and may result in the discontinuation of BCG treatment, adversely affecting oncological outcomes. Despite the considerable clinical impact of BCG-induced LUTS, the underlying mechanisms remain unclear, hindering the implementation or development of effective treatments. This review provides novel insights into the potential mechanisms underlying BCG-induced LUTS, focusing on the integrated roles of afferent and efferent nerves in both normal and pathological bladder sensation and function. Specifically, this review examines how the body’s response to BCG—through the development of inflammation, increased urothelial permeability, and altered urothelial signalling—might contribute to LUTS development. Drawing from known mechanisms in other common urological disorders and data from successful clinical trials involving NMIBC patients, this review summarises evidence supporting the likely changes in both sensory nerve signalling and bladder muscle function in the development of BCG-induced LUTS. However, further research is required to understand the intricate mechanisms underlying the development of BCG-induced LUTS and identify why some patients are more likely to experience BCG intolerance. Addressing these knowledge gaps could have profound implications for patients’ quality of life, treatment adherence, and overall outcomes in NMIBC care.

## 1. Introduction

### 1.1. Bladder Cancer

Bladder cancer is the 10th most prevalent cancer globally [1,2,3]. The risk of bladder cancer increases with age, with the incidence being double in white populations compared to African American populations [1,4]. Smoking is estimated to contribute to the development of 50% of bladder tumours and remains the most significant risk factor for patients [4,5].

Approximately 75% of bladder cancers are diagnosed as non-muscle invasive bladder cancer (NMIBC), while 25% present as muscle-invasive (MIBC) or metastatic disease [6]. NMIBCs are classified based on both the depth of tumour invasion and the histological presentation of the cancer cells as either carcinoma in situ (CIS), Ta, or T1 [7]. CIS and Ta tumours are confined exclusively to the superficial urothelial lining of the bladder whilst T1 tumours invade the subepithelial connective tissue, encompassing the lamina propria, but not the detrusor muscle [7]. MIBC is diagnosed when cancers infiltrate the detrusor muscle.

NMIBC is associated with a 5-year survival rate of 75–85% vs. 27–50% for MIBC [2,3,5]. However, NMIBC is associated with high recurrence (up to 80%), and approximately 40–50% of cases advance to MIBC [8]. MIBC is more lethal than NMIBC due to its higher likelihood of metastasising to lymph nodes and other organs [2,3,9]. Therefore, it is imperative that NMIBC is rapidly detected and treated prior to progression.

### 1.2. BCG Immunotherapy for NMIBC

Intravesical Bacillus Calmette–Guerin (BCG) immunotherapy is the most effective and ‘gold standard’ adjuvant treatment in intermediate-to-high-risk non-muscle invasive bladder cancer (NMIBC) patients following transurethral resection of the bladder tumour (TURBT) [10,11]. BCG is initially administered as an induction series of six once-weekly instillations and is typically followed by a BCG maintenance schedule for up to three years [12,13,14].

Following BCG instillation in the bladder, live BCG attaches to the urothelium via interactions between the fibronectin attachment protein on the BCG cell wall and the fibronectin present on tumour cells, leading to internalisation by urothelial cancer cells [15]. BCG adherence to the urothelium triggers an immune response within hours of instillation. This includes the release of an array of cytokines and chemokines from urothelial and antigen-presenting cells, which attract granulocytes and mononuclear cells to the bladder [16]. The immune response to BCG is characterised by granulomas in the bladder wall, consisting of clusters of macrophages, dendritic cells, lymphocytes, neutrophils, and fibroblasts [15,16,17]. This local migration of polymorphonuclear leukocytes into the tumour microenvironment is a crucial component of the therapeutic effect of BCG via the initiation of tumour cell death, although the specific mechanisms responsible have not yet been elucidated [11,12,15]. Repeated instillations during BCG induction enhance the immune response: the overall result is a significant and sustained localised immune response in the bladder urothelium and lamina propria that is crucial to its efficacy [11,12,15,18,19,20].

### 1.3. Adverse Effects of BCG Immunotherapy

BCG intravesical therapy is generally safe, with few patients experiencing serious systemic side effects that necessitate immediate discontinuation, including BCG dissemination to other sites via the bloodstream [21,22,23]. However, despite this positive safety profile, approximately 80% of patients develop significant BCG-related cystitis (bladder inflammation) [21,24,25,26]. BCG cystitis commonly appears within hours of BCG administration and typically co-occurs with the development of debilitating lower urinary tract symptoms (LUTS), including urinary urgency, urinary frequency, pelvic pain, and dysuria (painful urination) [23,27,28,29]. Mild-to-moderate BCG cystitis can be an indicator of an effective immune response, and the LUTS usually resolve within 48–72 h after each BCG infusion [30]. However, symptoms typically recur with each subsequent BCG instillation, and both the symptom severity and duration tend to increase with continued therapy, significantly impacting patients’ quality of life [21,23,30,31,32]. Prevention strategies, including a reduced BCG dose and shortened or modified BCG maintenance, can be utilised to keep patients on their BCG schedule [27] as persistent symptoms can have a significant impact on oncological outcomes. Up to 20% of patients discontinue BCG treatment due to the severity of their LUTS, most commonly within the first year. These patients are classified as BCG intolerant and have a significantly increased risk of cancer progression [24,33,34]. Limited effective treatment options are available for patients that are BCG intolerant, and radical cystectomy (complete bladder removal) is the primary recommended option [35]. However, radical cystectomy is associated with significant morbidity and has long-lasting impacts on patients’ quality of life [36,37]. Alternate intravesical treatment options include chemotherapy with Mitomycin C, Gemcitabine, and Anthracyclines such as Epirubicin [38]. The immune checkpoint inhibitor pembrolizumab [39] is used in advanced-stage clinical trials for NMIBC. However, whether these treatments offer advantageous side effect profiles compared to BCG is currently unknown.

Future therapeutic refinements in early-stage development include nanostructured vehicles (nano- and micro-scale) for intravesical drug delivery; these extend the retention time and enhance the permeability of BCG into the urothelium [40]. One such model system utilising a biotin–streptavidin strategy inhibits cancer progression and prolongs survival in pre-clinical rat/mouse models [41]. The impact of this delivery modification on side effects has not been explored and remains a long way from clinical translation.

Despite the known impact of BCG-induced side effects on patients’ quality of life and treatment adherence, there is no standard-of-care treatment to aid in the relief of BCG-induced LUTS. The development of effective adjunct therapies to treat or prevent BCG-induced LUTS has been limited by a lack of understanding of the mechanisms that drive these side effects. The objective of this review was to explore this significant knowledge gap and summarise the latest clinical and pre-clinical data to provide insights into the mechanisms that likely contribute to the development of BCG-induced LUTS. To achieve this, we performed a comprehensive literature search of major research repositories, and articles were selected for inclusion using existing expert knowledge of the mechanisms underlying the development of LUTS in a range of bladder disorders. We correlated the mechanisms known to underlie the development of LUTS in other common urinary disorders with the mechanisms underlying BCG efficacy, providing considerations for future research and clinical intervention. Understanding the mechanisms responsible for BCG-induced LUTS could have profound implications for NMIBC patients, providing an avenue for targeted adjunct therapies that will improve quality of life and treatment adherence.

## 2. Mechanisms Underlying the Development of LUTSs During BCG Immunotherapy

BCG-induced LUTS, including urinary urgency, urinary frequency, pelvic pain, and dysuria overlap considerably with the symptomology of a range of common bladder disorders including urinary tract infection (UTI), interstitial cystitis/bladder pain syndrome (IC/BPS), and overactive bladder (OAB) syndrome [42,43]. Whilst the mechanisms underlying the development of LUTS in UTI, IC/BPS and OAB have not been completely elucidated, numerous lines of evidence support changes in the excitability of bladder-innervating sensory (afferent) nerves and altered bladder contraction properties as crucial common factors [44,45]. Below we summarise the evidence that bladder afferent hypersensitivity may underlie the development of LUTS following BCG instillation, and the mechanisms that may drive the development of bladder afferent hypersensitivity during BCG immunotherapy for NMIBC.

### 2.1. Neural Control of Bladder Sensation and Function

Normal bladder function, characterised by urine storage and periodic urination (micturition), is achieved through the coordinated relaxation and contraction of the smooth muscle within the bladder wall and urethra, and the striated muscles of the outflow region and pelvic floor [46,47]. The coordination of bladder muscle function relies on the synchronised activity of the autonomic, sensory, and somatic nervous systems and has been comprehensively described in excellent reviews [47,48].

**Figure 1:**
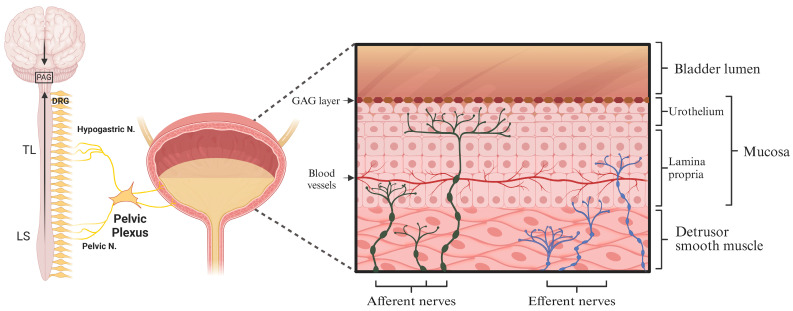
Structure and innervations of the bladder wall. The bladder is innervated by a complex network of sensory (afferent) and efferent nerves. Bladder afferent nerves terminate throughout the bladder wall, with endings located in the detrusor smooth muscle, lamina propria, and urothelium. As the bladder fills with urine, the bladder wall stretches, and mechanosensory afferent fibres embedded within the detrusor smooth muscle are activated. These afferent nerves, with cell bodies located in the dorsal root ganglia (DRG), travel through the pelvic and hypogastric/splanchnic nerves, synapsing in the dorsal horn of the lumbosacral (LS, L5-S1) and thoracolumbar (TL, T10-L2) regions of the spinal cord. Sensory signals arriving at the spinal cord synapse are transduced by second-order neurons to terminate in the periaqueductal gray (PAG), a brainstem hub for integrating sensory inputs from the spinal cord and descending input from higher brain centres. Changes in the excitability of bladder-innervating sensory nerves can thus directly impact bladder sensation and bladder function. Created in BioRender. Grundy, L. (2024) [53] www.BioRender.com/h79i418.

The bladder is innervated by a complex network of sensory (afferent) and efferent nerves [49,50]. Bladder afferent nerves terminate throughout the bladder wall, with endings located in the detrusor smooth muscle, lamina propria, and urothelium. These nerves are classified based on both their location and sensitivity to mechanical stimuli [42,51] (Figure 1). Bladder afferents located within the detrusor smooth muscle (muscular afferents) are highly sensitive to mechanical stretch (mechanosensory afferents), allowing for the detection of bladder fulness as the bladder wall stretches to accommodate increasing volumes of urine [52,53]. In contrast, bladder afferents terminating within the urothelium (mucosal afferents) are predominantly stretch insensitive [53,54]. Due to their lack of sensitivity to stretch, the primary stimulus from the bladder, the role of mucosal afferents in signalling during normal bladder function is unclear. However, it is proposed that the unique position of mucosal afferent endings close to the bladder lumen allows for an additional layer of sensory resolution, including the detection of changes in the bladder mucosal environment in response to infection, inflammation or the disruption of the urothelial barrier [53]. To achieve these diverse mechano- and chemo-sensitive roles, bladder afferents possess a variety of receptors and ion channels that integrate signals from this complex environment [53,54,55] and increase or decrease neuronal excitability to generate a variety of physiological and pathophysiological bladder sensations.

As the bladder fills with urine, the bladder wall stretches, and mechanosensory afferent fibres embedded within the detrusor smooth muscle are activated [52]. These afferent nerves, with cell bodies located in the dorsal root ganglia (DRG), travel through the pelvic and hypogastric/splanchnic nerves, synapsing in the dorsal horn of the lumbosacral (LS, L5-S1) and thoracolumbar (TL, T10-L2) regions of the spinal cord [47,54]. Sensory signals arrive at the spinal cord synapse and are transduced by second-order neurons to terminate in the periaqueductal gray (PAG), a brainstem hub for integrating sensory inputs from the spinal cord and descending input from higher brain centres [42,47,54] (Figure 1). When bladder volumes are low, bladder afferent signals are of low intensity and are responsible for activating central networks via the PAG that stimulate urine storage via the efferent inhibition of smooth muscle contraction in the bladder [56,57]. As the bladder continues to fill, afferent signals increase in intensity [52,54]. Once afferent signals increase above an individualised threshold, a conscious awareness of bladder fulness develops. At this point, switching from urine storage to voiding is dependent on a variety of psychological, situational, and social stimuli, which determine the appropriateness to urinate [47,48]. If permitted, the PAG receives appropriate cortical input and subsequently activates the pontine micturition centre (PMC) to stimulate parasympathetic efferent nerves to initiate the simultaneous contraction of the detrusor and the relaxation of the urethra to induce urination [47,48]. As a consequence, disruptions in the tightly regulated processes governing bladder afferent nerve excitability can have significant impacts on both bladder sensation and function and are considered central to the development of LUTS in various bladder disorders, including IC/BPS, OAB, and UTI [44,45,53]. The similarity in LUTS presentation after BCG immunotherapy and in other common bladder conditions suggests that shared underlying disruptions to the sensory pathways governing bladder sensation and function may contribute to the development of LUTS during BCG treatment for NMIBC.

### 2.2. Sensitisation of Bladder Afferent Nerves During Inflammation

Intravesical BCG, as described briefly above and in detail elsewhere, evokes a sustained and substantial immune response characterised by the recruitment of various immune cells and heightened levels of multiple cytokines [11,15]. Inflammation is an essential biological mechanism that serves as a protective response against a variety of threats, including infections and tissue damage [58]. In response to these threats inflammation increases, helping to marshal a protective response to promote infection clearance or tissue repair [59,60]. Inflammation is also associated with the development of exaggerated sensations such as pain and forms a crucial component of the host response to injury and infection by initiating protective behaviours [60,61,62]. Similarly, inflammation in the bladder manifests as heightened sensation, including urinary urgency, pelvic pain, dysuria, and exaggerated bladder function, which are highly prevalent in inflammatory bladder conditions such as IC/BPS, UTI and chemotherapy-induced cystitis [42,63]. As these symptoms overlap considerably with the symptoms of BCG cystitis, similar neuro-immune interactions may underlie the development of bladder symptoms following BCG immunotherapy for NMIBC, but these are yet to be explored.

**Figure 2:**
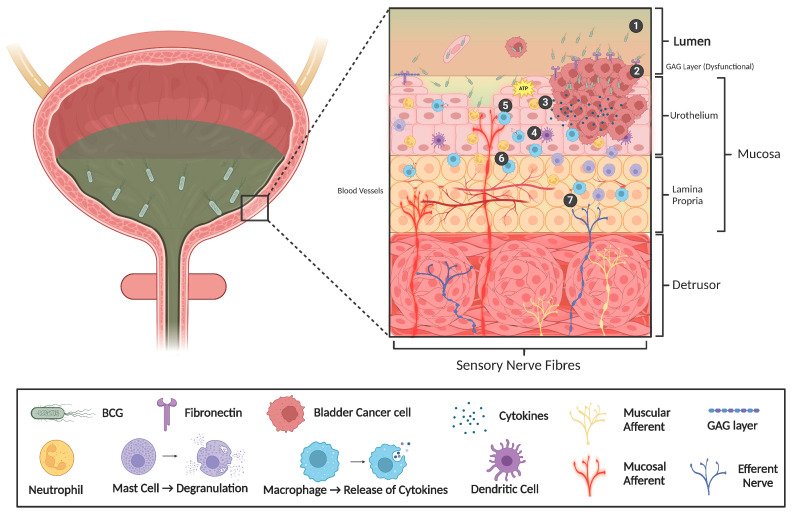
Proposed peripheral mechanisms underlying BCG-induced bladder hypersensitivity and dysfunction. Following BCG instillation into the bladder (1), live BCG attaches to the urothelium via the fibronectin present on tumour cells (2). BCG adherence to the urothelium triggers the release of cytokines and chemokines (3), stimulating immune cell infiltration into the urothelium (4) to initiate tumour cell death. Inflammation in the urothelium promotes GAG layer dysfunction and urothelial barrier breakdown (5), allowing urine, BCG, and commensal bacteria to reach the bladder interstitium. The urinary solutes and inflammatory mediators released from urothelial and immune cells have the potential to sensitise bladder afferent (6) and efferent (7) endings. Bladder afferent hypersensitivity increases peripheral drive to the spinal cord, leading to exaggerated bladder sensation and function. Exaggerated bladder sensation and function are proposed as key mechanisms underlying the development of LUTS. Created in BioRender. Grundy, L. (2024) [53] www.BioRender.com/a16d912.

A key mechanism underlying the development of inflammation-induced pathophysiological sensation is the sensitisation of sensory nerves to physiological stimuli [64,65,66,67]. In animal models of cystitis, where the direct interrogation of sensory signalling is possible and has been studied, animals exhibit bladder afferent hypersensitivity to distension and bladder dysfunction, providing a link between exaggerated signalling in the bladder and the development of a pathological phenotype [63]. Bladder afferents, like those of other organs, have chemo-sensitive properties, and can be sensitised directly by inflammatory mediators [44]. Cytokines, and mast cell mediators including histamine and nerve growth factor (NGF), can directly sensitise bladder afferents to induce hyperexcitability when exposed to physiological stimuli [68,69,70,71] and provoke hyperinnervation by sensory and sympathetic nerves [63,72,73]. NGF from mast cells can also promote antidromic neuropeptide secretion from peripheral afferent terminals, including substance P and calcitonin gene-related peptide, which contribute to neurogenic inflammation and the neuroplasticity of peripheral afferent circuits [42,74,75]. There are no studies exploring the changes in urinary NGF levels in BCG-induced cystitis; however, mast cells are present in NMIBC tumours [76,77] and are considered to play a key immunomodulatory role during BCG therapy [77]. BCG can also directly induce the release of cytokines from the bladder and urothelial cells in vitro [15,78,79], with many of these implicated in the sensitisation of bladder afferents [69] (Figure 2). Furthermore, the levels of histamine and inflammatory cytokines are elevated in the urine of patients undergoing BCG therapy [15,80,81], providing the fundamental basis for enhanced neuro-immune interactions during BCG immunotherapy. Despite these correlations, further research is required to determine whether BCG-induced inflammation can directly sensitise bladder afferents.

### 2.3. Altered Urothelial Permeability

The bladder and urinary tract are lined by the urothelium, one of the most impermeable transitional epithelium structures within the body. The urothelial barrier is crucial to the maintenance of bladder homeostasis, protecting the underlying interstitium of the bladder from the toxic waste metabolites and bacteria present in urine [82,83]. The urothelium comprises three cell layers: the basal layer, partially differentiated intermediate cells, and highly specialised apical cells [84,85]. Apical cells create a virtually impermeable barrier that is maintained by tight junctions, hydrophobic uroplakin plaques and a dense layer of glycosaminoglycan (GAG) on the apical surface that minimises the reabsorption of toxic urine components including ammonia, urea, potassium, and the attachment of bacteria to urothelial cells [82,86,87]. Damage to the bladder’s GAG layer, such that urothelial permeability increases, allows urine solutes to penetrate the bladder tissue and has been shown to directly sensitise bladder afferents [88] and contribute to the development of bladder pain and dysfunction in animal models of cystitis [89,90]. Furthermore, increased urothelial permeability promotes inflammation [91], which, as outlined above, can have profound impacts on bladder afferent excitability. Patients diagnosed with IC/BPS often exhibit diminished urothelial barrier integrity, which is characterized by the reduced expression of molecular markers of tight junction proteins [92,93,94,95]. This is considered a key factor in the development of lower urinary tract symptoms (LUTS) in IC/BPS patients [95].

Experimental evidence directly implicating BCG immunotherapy in urothelial barrier breakdown is limited; however, NMIBC patients undergoing BCG therapy have elevated levels of cytokines and albumin within the urine in the first 12 h after BCG treatment, indicating urothelial leakage [15,19]. Furthermore, intravesical BCG treatment causes the long-term downregulation of uroplakins in a mouse model, which is consistent with urothelial barrier dysfunction [96]. It is also important to consider that patients with NMIBC will undergo multiple cystoscopies that could damage the urothelium. Furthermore, TURBT alone will damage the integrity of the urothelial barrier and is a major reason for the break between TURBT and starting BCG therapy [97]. Another mechanism that likely impacts urothelial integrity during BCG immunotherapy is the development of chronic inflammation. The chronic inflammation observed in IC/PBS contributes to impaired urothelial homeostasis and abnormal function [98]. As such, if the inflammatory stimulus is persistent, inflammation and permeability can perpetuate each other. Therefore, as BCG immunotherapy induces an intense but persistent local inflammatory reaction in the bladder wall [15], BCG-induced inflammation may also promote an increase in urothelial permeability around the bladder tumour (Figure 2).

Supporting an increase in urothelial permeability as a contributing factor in the pathophysiology of BCG-induced LUTS, treatments that improve urothelial barrier function and are effective in treating LUTS in IC/PBS have also been utilised with some success in treating BCG-induced LUTS. Notably, agents such as Hyaluronic Acid (HA) and Chondroitin Sulfate (CS) [99,100] show clinical efficacy in treating LUTS following BCG immunotherapy [101,102,103]. The early repair of the GAG layer using intravesical HA and CS can significantly improve urinary urgency, bladder pain, frequency, and voided volume [101,104], indicating that increased urothelial permeability may represent a key mechanism underlying the development of BCG-induced LUTS (Figure 2).

### 2.4. Altered Urothelial Neurotransmission

In addition to functioning as an impermeable barrier, multiple lines of evidence indicate that the urothelium is a mechanosensory organ that can detect and respond to chemical and mechanical stimuli [105]. In response to stimulation, urothelial cells secrete a variety of signalling molecules including neurotrophins, neuropeptides, ATP, acetylcholine, prostaglandins, prostacyclin, nitric oxide, and cytokines, which enable communication with underlying afferent and efferent nerves, smooth muscle cells, interstitial cells, and inflammatory cells [105,106,107,108,109]. Several studies have implicated changes in urothelial mediator release and the development of LUTS in OAB and IC/PBS [108,109,110,111].

The primary method of communication in the urothelium is hypothesised to be via non-neuronal ATP, and its activation of bladder afferents nearby [112,113,114]. ATP is released from the urothelium in response to stretch, and enhanced ATP release from urothelial cells has been identified in IC/PBS patients and is correlated with painful sensations and bladder overactivity [108,109,115,116]. Furthermore, preclinical studies have demonstrated that chronic bladder inflammation increases ATP release from the urothelium and strengthens purinergic signalling in bladder afferents [117]. ATP is secreted during immunogenic cell death, and there is a correlation between inflammation and ATP release from epithelial cells, including the urothelium [117,118]. As BCG immunotherapy is associated with significant inflammation, as well as changes in the integrity of the urothelium, it is surprising that the altered release of urothelial neurotransmitters has not been explored in either humans or by utilising animal models of BCG treatment in the context of LUTS (Figure 2).

### 2.5. Altered Bladder Contractility

Bladder relaxation during urine storage and efficient bladder contraction during urine evacuation are essential for ensuring regular micturition. Bladder muscle function is under the control of efferent nerves, which can be influenced by the local bladder environment, as well as by the intensity of the afferent signal that feeds into the central circuits mediating efferent output [47,54]. Multiple lines of evidence support that increased detrusor contractions during the filling phase of micturition are a major component of the pathophysiology underlying the development of LUTS in OAB [119].

Research on the effects of BCG immunotherapy on bladder contractility is limited; however, some patients will experience urge incontinence during BCG infusion, contributing to BCG intolerance [120,121], indicating that BCG can rapidly impact bladder function in some patients. The mechanisms underlying this have yet to be explored, but the relative speed of the effect indicates either a reflex mechanism involving afferent nerves or direct local actions within the bladder wall [47,48] (Figure 2). Clinical studies have explored the contribution of exaggerated bladder function from an intervention perspective. Antimuscarinics are the mainstay of treatment for OAB; however, randomized controlled trials investigating the effectiveness of oxybutynin in treating BCG-induced LUTS are scarce and yield mixed results. A triple-blind, placebo-controlled study involving 60 patients receiving BCG infusions found that oxybutynin significantly alleviated BCG-induced LUTS, including urgency and dysuria [122]. However, a separate randomized trial of 50 BCG-naïve patients, who were administered oxybutynin alongside BCG infusions, showed increased urinary frequency and dysuria compared to those who received a placebo with BCG [123]. Mirabegron, a B_3_-adrenoreceptor agonist that provides relief for OAB through the increased relaxation of the bladder during filling [124], has been shown to improve LUTS in NMIBC patients undergoing BCG immunotherapy [125]. Specifically, mirabegron significantly improved the OAB symptom score, nocturia, micturition urgency, urinary incontinence, pain and patient compliance and prognosis [125], suggesting that increased bladder contraction or tone during bladder filling may be a fundamental mechanism underlying the development of LUTS following BCG immunotherapy for NMIBC. Botulinum neurotoxin type A (BoNT/A), a neurotoxin that blocks signalling at the neuromuscular junction [126] and is clinically effective in relieving OAB by reducing bladder contraction [127], has also been explored for relieving the side effects of BCG cystitis. Few studies have reported the positive effects of injecting BoNT/A into the bladder in patients with refractory BCG cystitis or BCG intolerance due to urge incontinence [128,129].

The mechanisms driving altered bladder muscle function during BCG immunotherapy are unknown, but a variety of biological processes that have been discussed in this review can influence the efferent regulation of bladder relaxation/contraction, including suburothelial inflammation, exaggerated afferent signalling, altered urothelial transmitter release, and heightened sensitivity to contraction-mediating transmitters. Further research is required to understand the relative importance of these complex interacting mechanisms in the context of the side effects of BCG.

## 3. Conclusions

BCG immunotherapy for NMIBC is commonly associated with the development of bladder side effects that can significantly impact patients’ quality of life and treatment adherence. Effectively managing or preventing these LUTS could significantly enhance patient well-being during and after NMIBC treatment, while also improving treatment compliance, particularly in BCG-intolerant patients. However, this review highlights that the development of such effective adjunct therapies is severely hindered by our limited understanding of the mechanisms behind BCG-induced LUTS. By exploring the similarities between other urological disorders and BCG-induced cystitis, this review provides crucial insights into a variety of underlying mechanisms that may contribute to BCG-immunotherapy-induced LUTS, including the development of exaggerated bladder sensory signalling, detrusor muscle hypercontractility, increased urothelial permeability, and altered urothelial signalling. Future research must focus on unravelling the specific role that these pathophysiological processes play in the development of LUTS following BCG immunotherapy. This knowledge could pave the way for developing adjunctive or modified therapies to complement BCG immunotherapy, potentially reducing the LUTS associated with NMIBC treatment to improve patients’ quality of life during treatment and treatment adherence.
